# Fate and transformation of silver nanoparticles in different biological conditions

**DOI:** 10.3762/bjnano.12.53

**Published:** 2021-07-07

**Authors:** Barbara Pem, Marija Ćurlin, Darija Domazet Jurašin, Valerije Vrček, Rinea Barbir, Vedran Micek, Raluca M Fratila, Jesus M de la Fuente, Ivana Vinković Vrček

**Affiliations:** 1 Institute for Medical Research and Occupational Health, Ksaverska cesta 2, 10 000 Zagreb, Croatia; 2 University of Zagreb, School of Medicine, Šalata 12, 10 000 Zagreb, Croatia; 3 Division of Physical Chemistry, Ruđer Bošković Institute, Bijenička cesta 54, 10 000 Zagreb, Croatia; 4 University of Zagreb, Faculty of Pharmacy and Biochemistry, Ante Kovačića 1, 10 000 Zagreb, Croatia; 5 Instituto de Nanociencia y Materiales de Aragón (INMA), CSIC-Universidad de Zaragoza, Zaragoza 50009, Spain; 6 Centro de Investigación Biomédica en Red de Bioingeniería, Biomateriales y Nanomedicina (CIBER-BBN), Spain

**Keywords:** animal tissue, biological media, nanoparticle aggregation, nanoparticle dissolution, nanoparticle reformation, silver nanoparticles

## Abstract

The exploitation of silver nanoparticles (AgNPs) in biomedicine represents more than one third of their overall application. Despite their wide use and significant amount of scientific data on their effects on biological systems, detailed insight into their in vivo fate is still lacking. This study aimed to elucidate the biotransformation patterns of AgNPs following oral administration. Colloidal stability, biochemical transformation, dissolution, and degradation behaviour of different types of AgNPs were evaluated in systems modelled to represent biological environments relevant for oral administration, as well as in cell culture media and tissue compartments obtained from animal models. A multimethod approach was employed by implementing light scattering (dynamic and electrophoretic) techniques, spectroscopy (UV–vis, atomic absorption, nuclear magnetic resonance) and transmission electron microscopy. The obtained results demonstrated that AgNPs may transform very quickly during their journey through different biological conditions. They are able to degrade to an ionic form and again reconstruct to a nanoparticulate form, depending on the biological environment determined by specific body compartments. As suggested for other inorganic nanoparticles by other research groups, AgNPs fail to preserve their specific integrity in in vivo settings.

## Introduction

The global consumption of silver nanoparticles (AgNPs) has been steadily increasing in the last decade and estimated to reach over 200 tons/year by 2022 [1]. AgNPs are extensively used in multiple industries and fields spanning from electronics, textiles, food, cosmetics to water treatment and healthcare [2–3]. A significant market contribution originates from the agriculture sector as evidenced by the Center for Food Safety, which listed more than 100 AgNP-containing food products [4]. The biomedical use of AgNPs represents the largest proportion of the market share [1] encompassing antimicrobial coatings on medical devices (catheters, stents, implants), wound dressings, targeted drug delivery, cancer therapy and diagnostics (biosensing, bioimaging) [2–3]. Such prevalence has raised concerns among the regulatory authorities about the safety of AgNPs for humans due to significant lack of relevant regulatory data. Thus, the Scientific Committee on Consumer Safety (SCCS) highlighted in its final Opinion on Colloidal Silver (nano) that insufficient data on AgNP physicochemical properties and toxicology in cosmetics hinder the health hazards caused by AgNPs [5]. Earlier, the Scientific Committee on Emerging and Newly Identified Health Risks (SCENIHR) raised questions on how different forms of Ag used in consumer and medical products may be related to human exposure and safety as AgNPs may undergo complex transformations in biological media [6].

It is well known that AgNP physicochemical characteristics, such as size, shape, surface charge, surface functionalization, or core composition determine their interactions with biological structures and affect their uptake, toxicokinetics, and toxicodynamics [7–14]. Therefore, any change in those properties will have consequences on the biological fate of AgNPs. No matter how well the properties of pristine AgNPs were tuned during production they will not be retained in biological media [9–14]. In biological media, AgNPs may be transformed into different forms by aggregation, agglomeration, dissolution, interaction with biomolecules, or generation of reactive oxygen species (ROS) that may lead to the coexistence of nanoparticulate, ionic, metallic, and complex salts forms [9–14]. Despite many scientific and medical evidences for (bio)chemical transformation patterns of AgNPs [15], questions about their final fate in the body are still open. Irreversible skin discoloration or argyria was described in patients that were overexposed to Ag via different routes [16–20]. In these patients, Ag granules were detected in the connective tissue of the dermis [18,20]. In fact, some Ag-containing drugs were retracted from clinical use due to the observed generalized argyria after long-term use or drug abuse [21]. Animal experiments demonstrated Ag accumulation in the liver, kidneys, brain, and testis after oral exposure to AgNPs; however, the chemical form of Ag remained undefined in these cases [22].

In media of high ionic strength and low pH the AgNPs aggregate and/or dissolve [23]. Under such conditions, the repulsive electrostatic forces between particles with the same surface charge are weakened, leading to aggregation upon collision [10]. The AgNPs with bulky coatings are less sensitive to this, since their stabilisation is steric and not electrostatic [14]. The dissolution of AgNPs is an oxidative process aided by protons [9,24–25] through which AgNPs release ions from the surface. The dissolution occurs faster for smaller NPs (larger surface area) and in the presence of molecules that may complex the ions [14]. Ag^+^ released into complex media is highly reactive and tends to associate with both organic and inorganic ions. With chlorides and sulphides, it forms AgCl and Ag_2_S, respectively, that are poorly soluble and precipitate into granules similar to aggregates [9–10,23–24]. The evaluation of AgNP biotransformation under in vitro settings revealed that argyrial deposits are created by several pathways, including partial AgNPs dissolution in the gastric fluid, uptake and systemic transport of ionic and nanoparticulate Ag as thiol and selenium complexes, and final deposition in the near-skin regions [15]. Especially important is the process of interaction with thiols owing to their many physiological roles and evidences for thiol-containing proteins as major targets for toxic effects of ionic Ag [25]. In the presence of biothiols the soluble complexes are formed. Thiols possess a high affinity for soft Lewis acid metals and may easily coordinate Ag^+^ [9,26–27]. The association of Ag with sulphur and the formation of Ag_2_S granules from ion exchange or Ag–thiol complexes is known as sulphidation [28–29].

However, many other scenarios should be considered during an in vivo journey of AgNPs and Ag^+^ ions, especially their interactions with biomolecules, such as lipids, metabolites, sugars, and proteins that will be adsorbed onto the nanoscale surface and spontaneously form biomolecular corona [9,11,25]. Corona generally protects from both aggregation and dissolution even though some high-affinity molecules might aid dissolution by pulling ions away from the surface [9,30].

Upon oral administration, AgNPs are first exposed to saliva and then to gastric fluid. These two media are characterized by different pH values, ranging from 6.2 to 7.6 in saliva or from 1.5 to 3.5 in gastric fluid, which may significantly affect AgNP dissolution. Moreover, the lining oral mucosa may significantly affect AgNPs and determine their colloidal stability and cellular interactions as evidenced earlier [27,31–33]. In the acidic medium of the stomach, AgNPs both agglomerate and dissolve [15,26,34]. The transformation will likely be incomplete due to protein corona formation and short residence time [9,15,26]. In the intestinal environment, a higher pH may slow down the loss of AgNPs [10]. Indeed, there is even evidence of agglomerates breaking down and releasing original AgNPs [32]. In both environments, the dissolved Ag may be released and may interact with many molecules. Although the majority of Ag will be cleared from the body through faeces [35], released Ag^+^ and its soluble complexes will be absorbed through passive or active transport [26]. It was also proposed that the AgNPs pass through the intestinal barrier [36–37]. Absorbed Ag will then interact with tissues and cells, being internalized through phagocytosis or pinocytosis and metabolised in lysosomes [11,26]. AgNPs will again dissolve in lysosomes due to their low pH and may be then released into the cytosol [38].

In short, the fate of AgNPs in the human body is extremely complex; however, many chemical pathways have not yet been systematically investigated and remain unclear. Here we aimed to examine the biotransformation of differently coated AgNPs [39] simulating real body conditions after oral uptake. The systematic work presented here addresses not only the evolution of various types of AgNPs in different media and tissue extracts, but also demonstrates how different AgNPs may be affected in biological media in a short time leading to the formation of new types of materials in different tissues. The reduction and de novo synthesis of AgNPs in different biological matrices should be considered during a risk assessment of AgNP-based consumer products.

## Results and Discussion

The fate of metallic NPs in the human body is a critical question for assessing their safety and efficacy when used in different medical or consumer products. Due to technical limitations and, more importantly, ethical constraints, it is usually not possible to track NPs in human tissues, which demands the implementation of biological model systems either in vitro or in vivo. This study was motivated by an interesting observation that emerged already during our previous research on acute effects of AgNPs in rodents [40]. Here, we exposed Wistar rats to poly(vinylpyrrolidone) (PVP)-coated AgNPs for 28 days. A microscopic examination of liver tissues of treated animals revealed an interesting result (described below) that initiated our subsequent research (presented here) on a possible transformation pattern of AgNPs in different biological environments during their in vivo journey. The colloidal stability, size, charge, and dissolution behaviour of AgNPs stabilized with neutral, positively, and negatively charged coating agents were determined after incubation in artificial media (depicted in Table 1) as well as in real biological fluids, obtained from animal experiments. A multimethod approach was used to examine their behaviour and transformation under experimental conditions relevant for in vivo settings by performing dynamic light scattering (DLS), electrophoretic light scattering (ELS), graphite furnace atomic absorption spectroscopy (GF-AAS), nuclear magnetic resonance (NMR) spectroscopy, and transmission electron microscopy (TEM) experiments.

**Table 1:** Information on the composition and pH of biologically relevant artificial media used for the evaluation of the stability and transformation of AgNPs.

abbreviation	medium	pH	composition

CCM	high glucose Dulbecco’s modified Eagle medium (DMEM)	7.2	commercial (containing 4500 mg/L glucose, sodium pyruvate, and sodium bicarbonate, without ʟ-glutamine)
m(CCM+BSA)	DMEM + 10% bovine serum albumin (BSA)	7.2	commercial (containing 4500 mg/L glucose, sodium pyruvate, and sodium bicarbonate, without ʟ-glutamine)
mCYS	cysteine solution	5.3	100 mg/L cysteine
m(CYS+BSA)	cysteine solution with BSA	6.4	100 mg/L cysteine + 500 mg/L BSA
mGSH	glutathione solution	3.2	100 mg/L glutathione
m(GSH+BSA)	glutathione solution with BSA	4.7	100 mg/L glutathione + 500 mg/L BSA
ALF	artificial lysosomal fluid	4.5	NaCl (3.210 g/L), NaOH (6.000 g/L), citric acid (20.800 g/L), CaCl_2_ (0.097 g/L), NaH_2_PO_4_∙7H_2_O (0.179 g/L), Na_2_SO_4_ (0.039 g/L), MgCl_2_∙H_2_O (0.106 g/L), glycerol (0.059 g/L), sodium citrate dihydrate (0.077 g/L), sodium tartrate dihydrate (0.090 g/L), sodium lactate (0.085 g/L), sodium pyruvate (0.086 g/L), formaldehyde (1.000 mL/L)
AGF	artificial gastric fluid	2.0	NaCl (34.2 mM), pepsin (0.1 g/L), HCl q.s. pH 2
PBS	phosphate buffer saline	7.4	KH_2_PO_4_ (0.144 g/L), Na_2_HPO_4_∙7H_2_O (0.795 g/L), NaCl (9 g/L)

### Physicochemical characteristics of freshly prepared AgNPs

**Figure 1:**
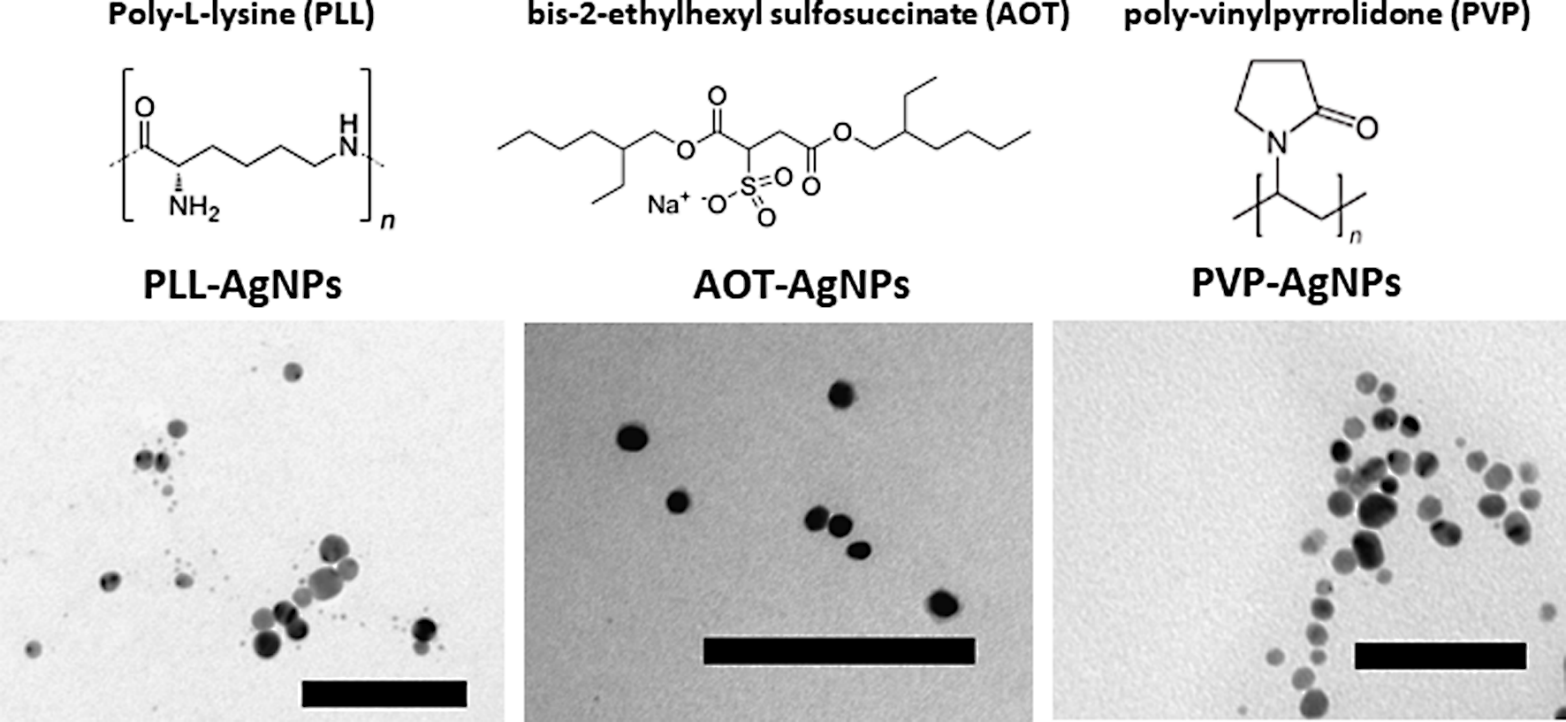
TEM images of freshly synthesized silver nanoparticles (AgNPs) coated with PLL, AOT, or PVP dispersed in ultrapure water at a concentration of 10 mg Ag/L. Scale bars are 100 nm.

Freshly prepared AgNPs coated with PVP, sodium bis(2-ethylhexyl)sulfosuccinate (AOT), and poly(ʟ-lysine) (PLL) were dispersed in ultrapure water (UPW) and examined by TEM to confirm their original morphology. These coating agents were selected according to their relevance in biomedicine, as they are the most frequently used stabilization agents for AgNPs according to the Web of Science database [41]. Moreover, they cover neutral, positive, and negative charge. All three AgNPs were found to be spherical, with a primary size below 10 nm (Figure 1). They were further examined by DLS and ELS (Table 2). The hydrodynamic diameter (*d*_H_) of all three AgNPs was below 10 nm, matching the primary size, with a small percentage of 20 nm particles in the case of AOT- and PVP-coated AgNPs. The major difference between different AgNPs was their potential values ζ, which were consistent with the charges of coating molecules used for their decoration. The ζ potential of AOT-AgNPs was strongly negative (−35.8 ± 6.9 mV), while for PLL-AgNPs it was strongly positive (+44.9 ± 1.4 mV). As a consequence, both AgNPs exhibited a long-term (over 2 months) stability in UPW due to electrostatic repulsive forces. Indeed, the NPs are considered electrostatically stabilised if their ζ potential is above +30 mV or below −30 mV [42]. However, PVP-AgNPs were weakly negatively charged, even though PVP is a neutral polymer. The reason behind this can be found in residual BH_4_^−^ ions that became strongly attached to the surface during the synthetic process and could not be removed by purification. Despite the ζ potential of −24.3 ± 3.7 mV, PVP provided good colloidal stability through steric stabilisation.

**Table 2:** Values of primary size (obtained by TEM), hydrodynamic diameter (*d*_H_), zeta potential (ζ), and percentages of released ionic silver in colloidal suspension of AgNPs coated with PLL, AOT, or PVP.

nanoparticle	primary size (nm)	*d*_H_ (nm)	ζ (mV)	%Ag^+^

PLL-AgNP	8.4 ± 4.7	4.6 ± 0.9 (97%), 15.2 ± 5.3 (3%)	44.9 ± 6.5	0.8
PVP-AgNP	9.2 ± 3.6	5.0 ± 1.2 (98%), 31.2 ± 3.5 (2%)	−24.3 ± 3.7	0.6
AOT-AgNP	7.8 ± 4.4	8.5 ± 4.5 (100%)	−35.8 ± 6.9	0.4

The amount of free Ag^+^ ions in AgNPs dispersed in UPW at a concentration of 10 mg Ag/L was determined to be below 1% for all the three cases, confirming the coating protection against dissolution.

### Experiments with animal tissues

**Figure 2:**
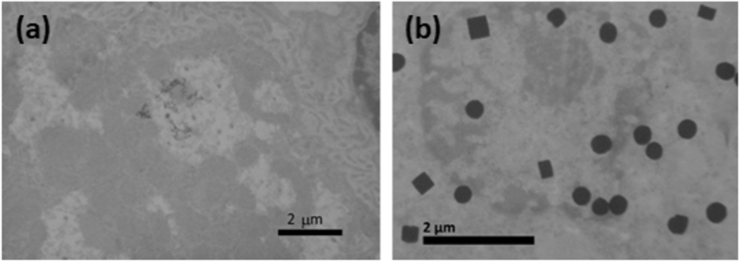
TEM images of liver obtained from (a) untreated and (b) treated healthy male Wistar rats. The rats were treated orally with a daily dose of 1 mg Ag/kg of body weight of PVP-coated AgNPs during 28 days.

In animal experiments, PVP-coated AgNPs were given orally to three-month old male Wistar rats of 320–350 g of body weight (b.w.) at a daily dose of 1 mg Ag/kg b.w. After 28 days of exposure, the rats were sacrificed under general anaesthesia and tissues were collected for further analysis. This may be regarded as an extension of our previous work [40]. In addition, liver tissues of control (untreated) and treated animals were prepared for TEM analysis, which revealed quite surprising observations. While TEM images obtained for control animals conformed to classical histological features of liver tissue (Figure 2a), large electron-dense spherical and cubic forms were found in the liver slices of treated animals (Figure 2b).

**Figure 3:**
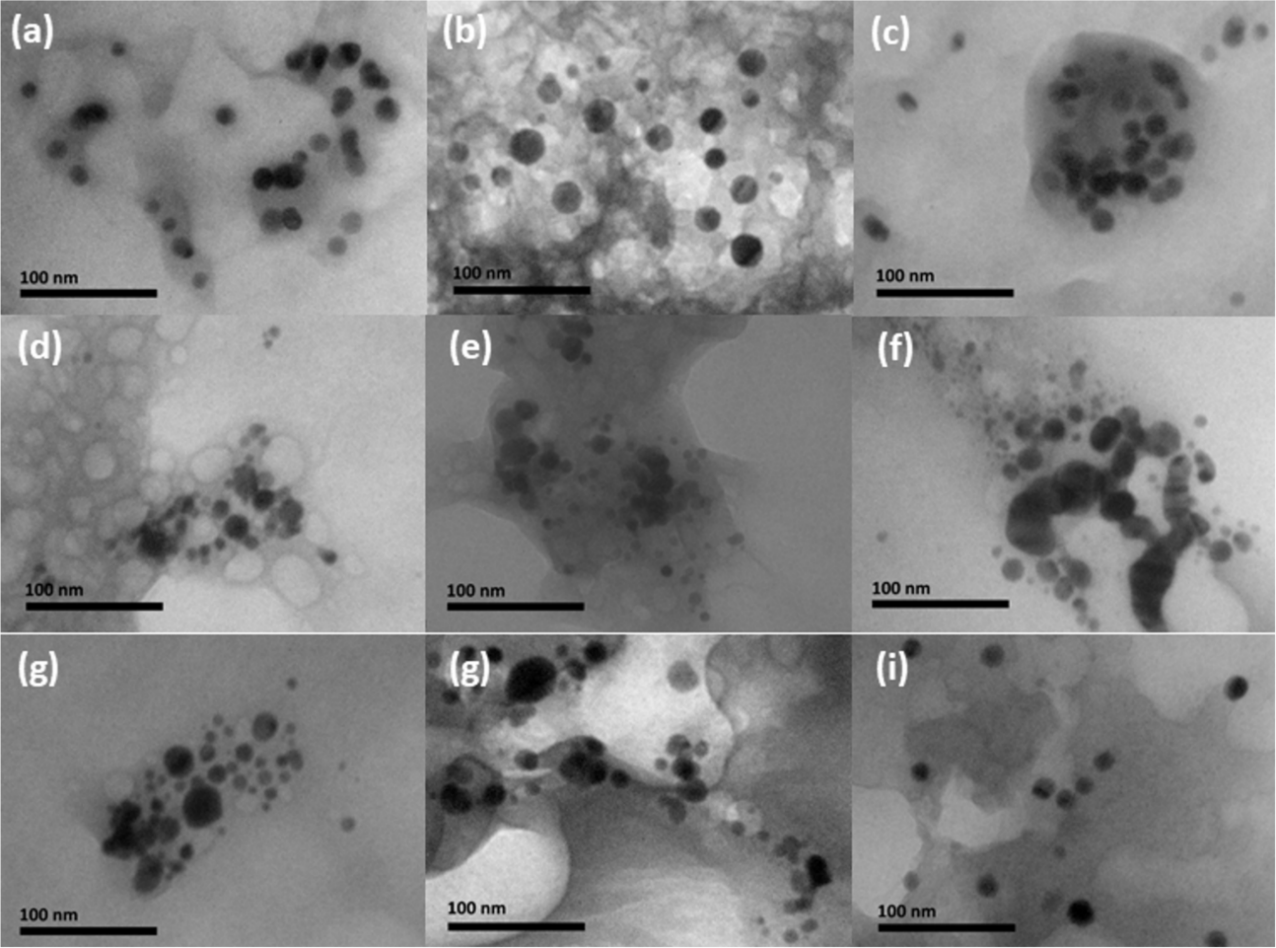
TEM images of differently coated AgNPs incubated for 30 min in 1% (w/v) tissue homogenates at a concentration of 100 mg Ag/L. (a) PVP-AgNPs (kidney), (b) PVP-AgNPs (liver), (c) PVP-AgNPs (brain), (d) AOT-AgNPs (kidney), (e) AOT-AgNPs (liver), (f) AOT-AgNPs (brain), (g) PLL-AgNPs (kidney), (h) PLL-AgNPs (liver), (i) PLL-AgNPs (brain). Scale bars are 100 nm.

**Figure 4:**
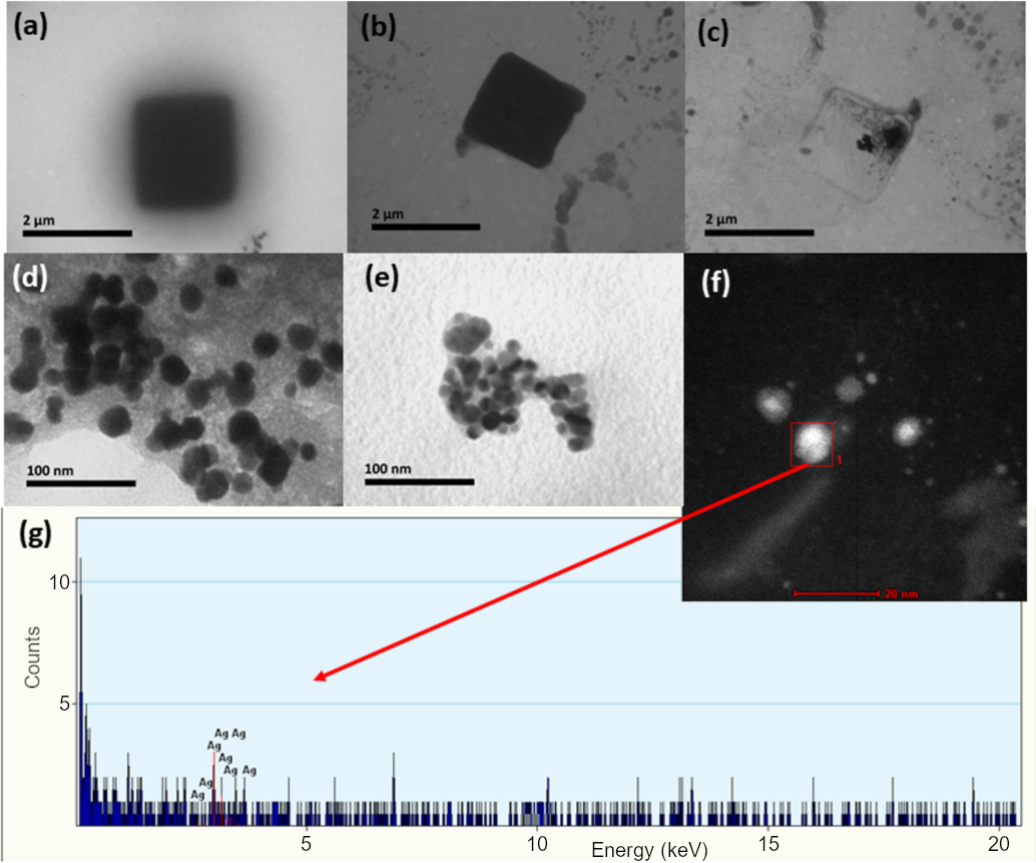
TEM images of objects observed after 30 min of incubation of AgNPs or Ag^+^ in different test media at a concentration of 100 mg Ag/L. (a) PLL-AgNPs in 1% (w/v) of blood plasma, (b) PLL-AgNPs in PBS, (c) electron-beam-induced transformation of cubic nanocrystals formed after incubation of AgNPs in PBS, (d) AgNPs formed after Ag^+^ incubation in 1% (w/v) of liver homogenate, (e) AgNPs formed after Ag^+^ incubation in 1% (w/v) of brain homogenate, (f) HAADF images of AgNPs formed after incubation of Ag^+^ in brain homogenate, (g) EDX analysis of nanoscale objects found in brain homogenates after incubation with AgNO_3_.

Our first explanation for the origin of these forms was the preparation of the tissue samples for TEM examination (i.e., precipitates of Pb acetate or OsO_4_). However, such electron-dense cubic or spherical structures were not found in the liver of control animals, meaning that they originate from the accumulation and biotransformation of AgNPs. Indeed, Prucek et al. [43] reported rapid crystallization of primary AgNPs to a one-order larger crystals in NaCl-rich media, in which a high concentration of chloride ions catalyse a controlled recrystallization of AgNPs. They observed structures similar to the forms presented in Figure 2b. As these large cubic and ball-like structures were not located inside hepatocytes, but extracellularly, we concluded that the crystallization of PVP-AgNPs, as observed in Figure 2b, occurred already in the circulation as blood represents a chloride-rich medium. To gain more detailed insight into the biotransformation of AgNPs, differently coated AgNPs were incubated for 30 min at a concentration of 100 mg Ag/L in PBS, whole blood (WB), blood plasma (BP), or in 1% (w/v) liver, brain, and kidney homogenates. In most tissue homogenates, AgNPs were well dispersed as shown in Figure 3. Although some aggregates were also visible under these conditions, there were no large crystals in kidney, liver, or brain homogenates as observed in vivo (Figure 2). However, the formation of large crystals was observed after incubation of AgNPs in PBS or BP (Figure 4). These crystal objects, with sizes in the μm range, were totally destructed after electron radiation in TEM (Figure 4c), similar to those observed by Prucek et al. [43].

Nanoscale objects found in PBS and BP incubated with different AgNPs (Figure 4) may, thus, be an indirect evidence of AgCl crystal formation in PBS and BP, which is possible only if the ionic Ag form is released form the AgNP surface by an oxidative etching process. Such release may be accelerated in the presence of complexing agents [44–45]. Henglein et al. [46] suggested the mechanism of coordination of Ag^+^ on the nanoscale surface by halide ions in media where halide ions are present at a high concentration. Formed AgCl_2_^−^ complex ions then undergo recrystallization into large objects similar to those observed in PBS and BP media. We tried to quantify the release of Ag^+^ from the surface of different AgNPs incubated in WB or tissue homogenates. However, all the attempts to extract free Ag^+^ ions from such media by ultracentrifuge filtration failed. Probably, free Ag^+^ ions were either bound to biomolecules or embedded in crystals that were unable to pass through 3 kDa pores of ultracentrifuge filters (see Experimental section). Due to these experimental hurdles, we performed an additional evaluation of the fate of the ionic Ag form in biological media, such as WB and tissue homogenates. An examination of the TEM data revealed quite interesting results. Incubation of AgNO_3_ in the liver and brain homogenates led to the formation of small AgNPs as presented in Figure 4d and Figure 4e, which was confirmed by energy-dispersive X-ray spectroscopy (EDX) (Figure 4f and Figure 4g).

### Transformation of different AgNPs in artificial biological media

**Figure 5:**
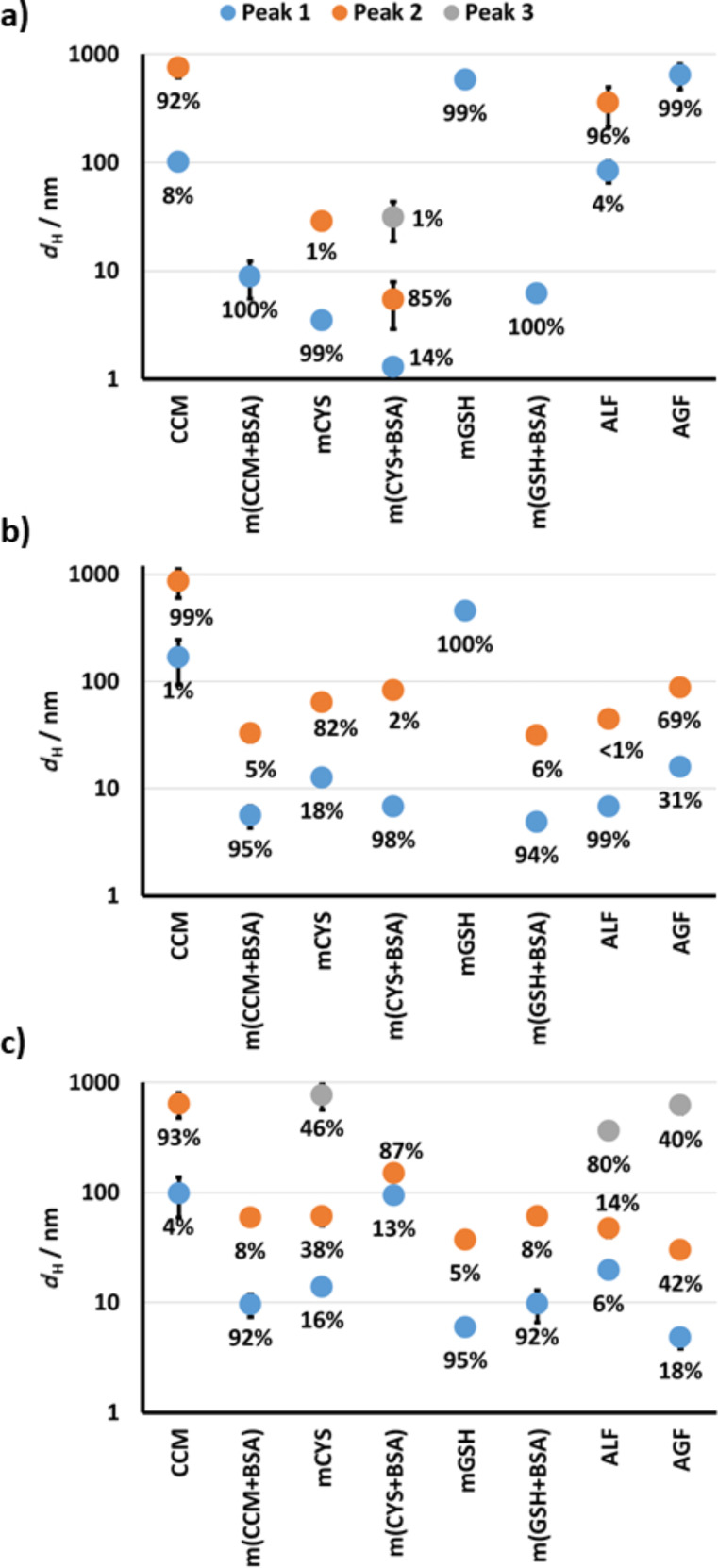
Changes in the hydrodynamic diameter (*d*_H_) of (a) AOT-AgNPs, (b) PVP-AgNPs, and (c) PLL-AgNPs in different biological media after 24 h of incubation. The media abbreviations are explained in Table 1. The existence of two or three peaks indicates a bimodal or trimodal size distribution, respectively, with the volume percentage given next to each point.

**Figure 6:**
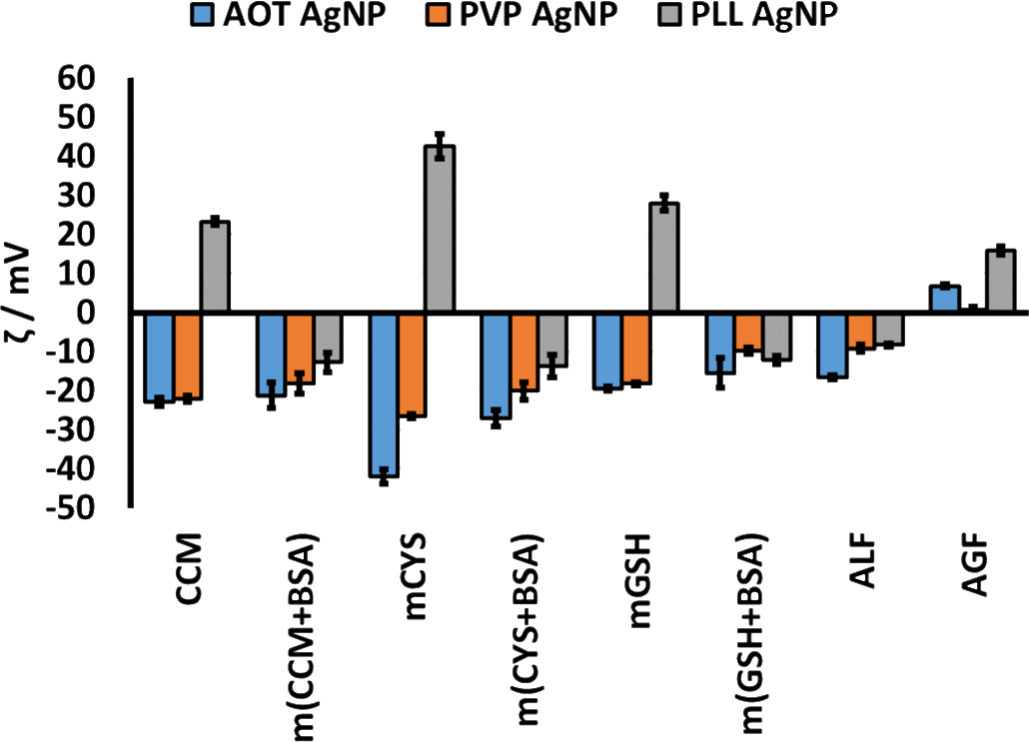
Changes in the *ζ* potential (in mV) of AOT-AgNPs, PVP-AgNPs, and PLL-AgNPs in different artificial biological media after 24 h of incubation. The media abbreviations are explained in Table 1.

In order to gain more coherent insight into the transformation of different AgNPs in biological media, we examined changes in their size, surface charge, and dissolution in simpler media (i.e., CCM, m(CCM+BSA), mCYS, m(CYS+BSA), mGSH, m(GSH+BSA), ALF, and AGF, see Table 1) after 0, 1, 4, and 24 h of incubation. The media AGF, ALF, and CCM can be used to evaluate the AgNP transformation during their passage from the gastrointestinal system into the body, after entering the extracellular matrix and going through cellular uptake. Furthermore, the behaviour of AgNPs was studied in the presence of the most relevant biothiols: cysteine (CYS) and glutathione (GSH). Data on the size distribution of different AgNPs in the tested media are given in Figure 5 and in Supporting Information File 1, Table S1, while observed ζ potentials are presented in Figure 6 and in Supporting Information File 1, Table S2. Although differences in the agglomeration behaviour among different AgNPs were observed depending on the dispersion media, an increase in agglomeration was generally observed in media with a higher ionic strength (CCM, mCYS, mGSH, ALF, and AGF, see Table 1). Such behaviour can be attributed to the loss of electrostatic repulsion between particles due to the complexation with counter ions present in media with high ionic strength [8,47]. The presence of proteins prevented AgNP agglomeration in m(CCM+BSA), m(CYS+BSA), m(GSH+BSA) due to the formation of protein corona on the surface of AgNPs even in media with a high ionic strength and low pH [8,48–49]. The ELS measurements (Figure 6 and Supporting Information File 1, Table S2) confirmed this as all AgNPs in the presence of BSA were characterized by a ζ potential close to the value observed for BSA (−7.6 mV). In CCM with and without the addition of BSA as well as in m(GSH+BSA), all AgNPs showed the same behaviour. Negatively charged AgNPs (AOT- and PVP-coated) agglomerated less in CYS-containing media than positively charged PLL-AgNPs, while an opposite trend was observed in the mGSH medium. This was likely a result of other mechanisms, possibly a direct interaction of CYS and PLL or due to a well-established cross-linking property of CYS [50–51]. This effect appeared to be so strong that it counteracted the stabilising power of BSA for PLL-AgNPs in m(CYS+BSA).

The DLS results indicated that mGSH did not induce a significant agglomeration of PLL-AgNPs (Figure 5) probably due to the supportive complexation of negatively charged GSH to the positively charged PLL, which was evident at a much lower ζ potential value of PLL-AgNPs in mGSH (Figure 6) compared to UPW (Table 2). Obviously, the complexation power of GSH was decreased in the case of PVP and AOT coating agents due to either unfavourable steric or electrostatic interactions, respectively. In both ALF and AGF, a significant agglomeration occurred for all three types of AgNPs. Moreover, a significant precipitation, visible in the test vials already after 1 h, prevented the DLS instrument to measure large agglomerates of PVP-AgNPs in ALF and AGF as well as that of PLL-AgNPs in AGF. The colloidal instability of all tested AgNPs was also evidenced by a significant decrease in the absolute value of their ζ potential in acidic AGF and ALF media (Figure 6). The precipitates observed in AGF and ALF media, both rich in chloride ions, probably originate from AgCl that can be formed from released Ag^+^ after the stripping of coating agents from the nanoscale surface which is prone to dissolution [10]. However, the speciation of Ag forms in biological systems is highly intricate as the formation of a wide range of Ag complexes is possible including those with biothiols and chloride [25].

### The fate of released Ag^+^ in the presence of GSH

In almost all tested media, the degradation of AgNPs leading to the release of free Ag^+^ ions was assumed. Indeed, any interaction between the nanoscale surface with complexing agents, such as biothiols, the dispersion of AgNPs into acidic or chloride-rich media, oxidative actions on the nanoscale surface mediated by ROS or catalysed by biomolecules may lead to Ag^+^ release [25]. Moreover, AgNP degradation and dissolution may occur after oral intake in different biological compartments including gastric and lysosomal fluid. The dissolution of tested AgNPs in different artificial biological media was evaluated by ultracentrifugation combined with GF-AAS. The dissolution data (given in Supporting Information File 1, Table S3) revealed that the released Ag^+^ fraction did not exceed 3% (w/v) of total Ag content. These results cannot be taken as evidence of AgNP stability and they do not necessarily contradict previous findings. All artificial media contain substances which can precipitate Ag^+^, either in the form of complexes similar to those with CYS and GSH or as insoluble salts, such as AgCl. Such precipitates cannot pass through the filter used here to separate free Ag^+^ ions, leading to the false negative reading of released Ag fraction. Moreover, CYS or GSH may reduce Ag^+^ ions back into AgNPs [15].

**Figure 7:**
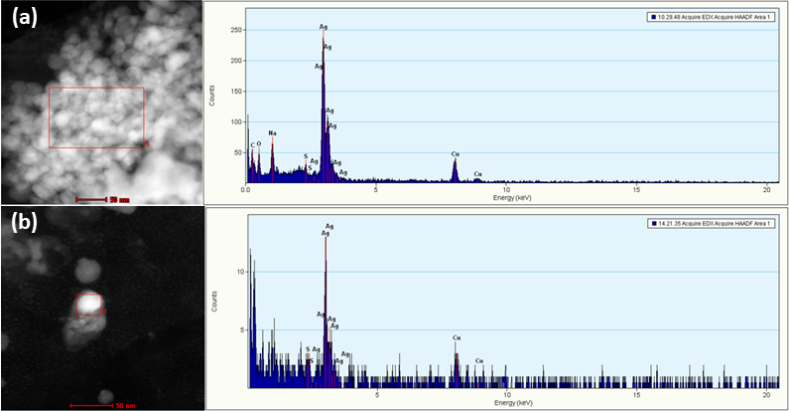
HAADF–STEM images of the newly formed AgNPs in (a) mCYS and (b) mGSH media. The media abbreviations are explained in Table 1. The areas identified by red rectangles represent areas examined by EDX. The corresponding EDX spectra are given next to the respective HAADF–STEM images.

The de novo formation of AgNPs from primary particles or AgNO_3_ was shown in cellular fractions [52]. Also, biogenic synthesis was noted in bacterial cells and in many other organisms. It stands to reason that human intracellular or extracellular environments can provide similar conditions [53–54]. Secondary particles are often found to contain Ag, S, and Cl [34]. Indeed, evidences on the formation of small AgNPs after only 30 min of incubation of AgNO_3_ in 1% (w/v) brain and liver homogenates is presented above (see Figure 4f and Figure 4g). In addition, we tested such scenario by dissolving AgNO_3_ into mCYS and mGSH media. Similar to tissue homogenates, small AgNPs were again found by TEM examination (Figure 7). The elemental mapping of newly formed particles was done via EDX. The EDX spectra (Figure 7c) showed Ag, S, Na, C, O, and Cu. The signals corresponding to C, O, and Cu resulted from the carbon copper grid, while the Ag signal evidenced that the captured particles were generated from AgNPs by the reductive power of CYS (Figure 7a) or GSH (Figure 7b).

**Figure 8:**
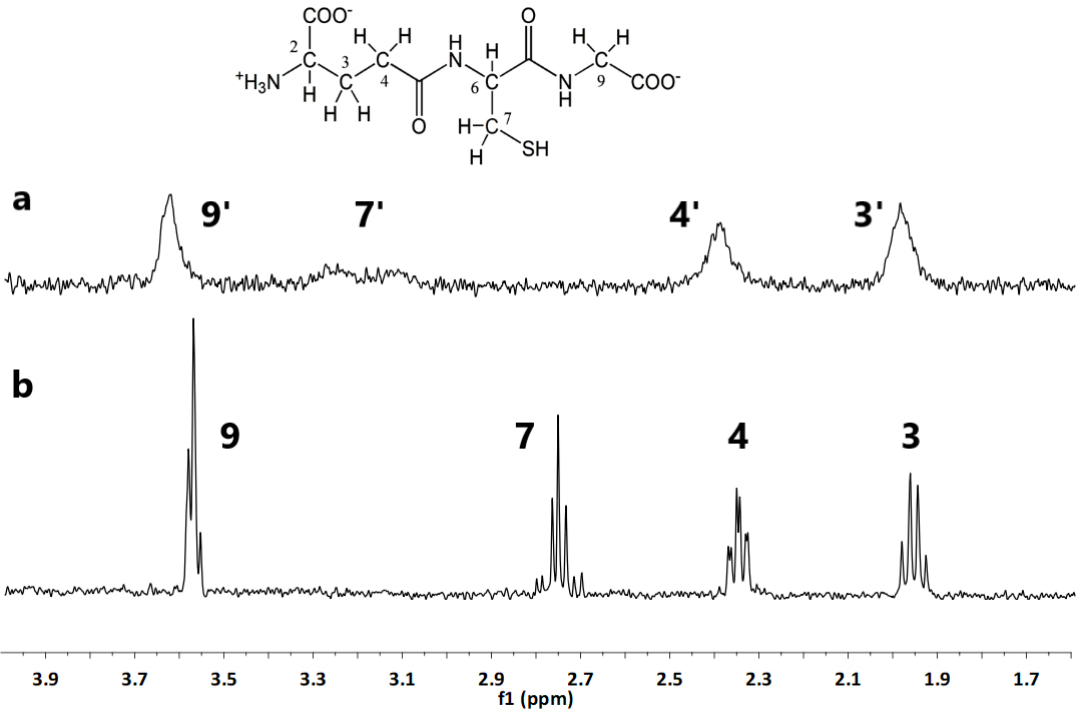
^1^H NMR spectra of (a) GSH-coated AgNPs formed during the interaction between AgNO_3_ and GSH and (b) free GSH. Both spectra are obtained in 25 mM phosphate buffer (pH 6.8). The structure of reduced GSH is shown above panel (a).

Finally, the fate of GSH during the formation of AgNPs was monitored by ^1^H NMR spectroscopy (Figure 8). The reaction was conducted in 25 mM of PB to maintain the pH close to neutral, since the synthesis does not progress in water due to the acidic nature of GSH.

The ^1^H NMR spectrum of 10 mM GSH in PB was recorded (Figure 8b). It showed four distinct peaks corresponding to protons bound to carbon at positions nine (overlapped with two), seven, four, and three. The proton at position six overlapped with the solvent signal and thus is not shown. The peaks were assigned according to AIST Spectral Database for Organic Compounds [55]. Then, a 5 mM solution of AgNO_3_ was added and the mixture was left to react for 2 h. During that time, the solution became opaque and changed colour to brownish-grey indicating the formation of small AgNPs. Afterwards, the NMR spectrum was recorded again (Figure 8a). All peaks shifted downfield by 0.5 ppm, and the peak seven split into two, which indicates that some interaction was happening through the thiol group (also bound to C7).

This possibly implies the formation of oxidised glutathione (GSSG), as noted in our previous work [56]. The most significant changes observed were the broadening of the peaks and loss of resolution, both of which are known signs of ligand binding to the NP surface [57–59]. Therefore, the spectral evidence points to the generation of AgNPs with GSH as both the reducing and coating agent, which likely binds to the surface in its oxidised form. Similarly, Ag cluster nucleation finally leading to AgNP formation was observed also for the interaction of Ag^+^ with CYS [60]. Similar to our previous NMR observation on the CYS oxidation to cystine during Ag cluster nucleation [60], the experiments presented here also indicate the conversion of GSH to GSSG during the nucleation of Ag clusters with subsequent AgNP synthesis.

## Conclusion

**Figure 9:**
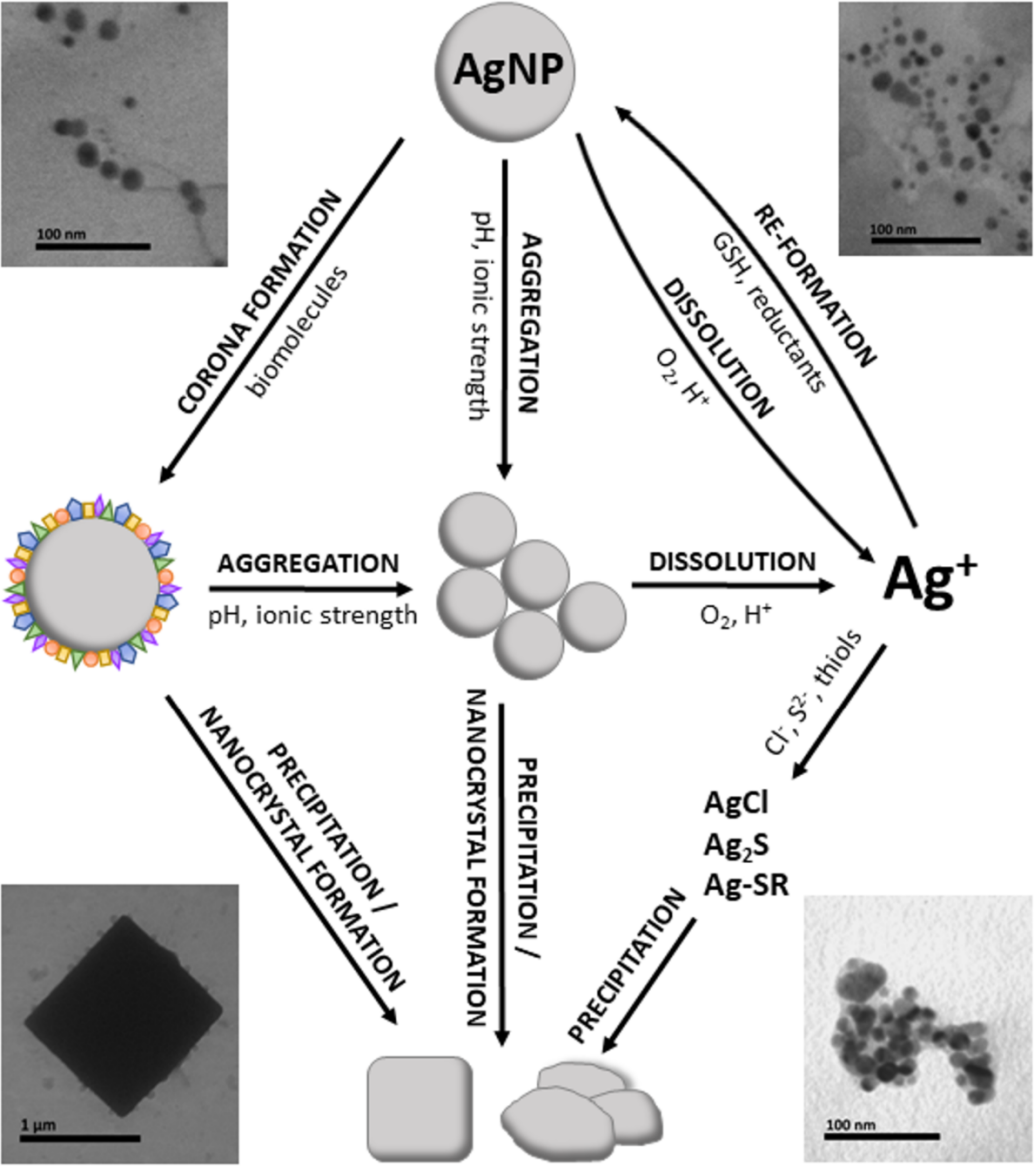
Biotransformation patterns of AgNPs in biological media.

The evaluation of NP behaviour in biological media is a crucial step toward safe biomedical applications. The biotransformation of AgNPs in the human body results in loss of integrity of AgNPs. Changes in AgNPs include aggregation, dissolution, and degradation which lead to the de novo formation of crystals in different tissues (Figure 9). Initially, primary AgNPs enter the body where they gain a protein corona, aggregate, and dissolve to Ag^+^. Ionic silver may precipitate in the anion-rich environment of different tissues, where Ag binds to S, resulting in nanocrystals. Aggregation and corona–NP destabilisation can also lead to precipitation. Whole blood, liver, and kidney are glutathione-rich environments, where GSH serves as an in vivo reducing agent for Ag^+^ converting it into secondary particles.

In simple biological media, an increase in the agglomeration of different AgNPs was generally observed with an increase in ionic strength due to the complexation of particles with counter ions present in media and loss of electrostatic repulsion between particles. However, the formation of protein corona prevented such agglomeration even in media with high ionic strength and low pH. Negatively charged AgNPs agglomerated less in CYS-containing media compared to positively charged PLL-AgNPs possibly due to the cross-linking property of CYS. Negatively charged GSH interacted with positively charged PLL-AgNPs leading to their agglomeration, while the GSH complexation power was decreased for PVP- and AOT-coated AgNPs due to unfavourable steric or electrostatic interactions, respectively. In most tissue homogenates, AgNPs were well dispersed; however, the crystallization of AgNPs was observed in chloride-rich media resulting in the formation of large cubic and ball-like crystals as an indirect evidence of the formation of AgCl crystals and oxidative etching process of AgNPs. The reformation of AgNPs from primary particles or released Ag^+^ was evidenced by the incubation of AgNO_3_ in liver and brain homogenates which led to the formation of small AgNPs. Moreover, NMR experiments demonstrated the crucial role of biothiols in this reformation process.

Our results demonstrated that the transformation pathway after the exposure of AgNPs to biological environments is highly dependent on the properties of the media, including ionic strength and the presence of proteins. These findings contribute to the understanding of the in vivo fate of AgNPs and the possible toxic effects following the biomedical applications.

## Experimental

### Chemicals

All chemicals were purchased from Merck (Darmstadt, Germany) unless otherwise specified. Ultrapure water (UPW), characterized with conductivity of 18.2 MΩ·cm, was obtained from a GenPure UltraPure water system (GenPure UV, TKA Wasseraufbereitungssysteme GmbH, Niederelbert, Germany).

### Synthesis and characterization of AgNPs stabilized with different coating agents

AgNPs were synthesised by the reduction of silver nitrate with sodium borohydride in the presence of AOT, PVP, and PLL as coating agents to provide colloidal stability to different AgNPs. The detailed procedure was described previously [39]. The final concentrations of the reagents in the reaction mixture were 0.5, 0.075, and 0.02 mM for AOT, PVP, PLL, respectively, while AgNO_3_ and NaBH_4_ were added to the final concentration of 2.3 and 5 mM, respectively.

The synthesis of AgNPs in the presence of GSH was performed by mixing appropriate amounts of AgNO_3_ and GSH to the final concentrations of 20 and 10 mM, respectively, in 25 mM of phosphate buffer solution (K_2_HPO_4_/KH_2_PO_4_, 0.401:0.014, w/w [g]). The reaction mixture was left to react for 2 h at room temperature, under constant stirring. The final pH of the reaction mixture was 6.8.

After synthesis, the AgNP suspensions were centrifuged twice at 15,000*g* for 40 min, resuspended in UPW and kept in the dark at 4 °C. The concentration of Ag in AgNP suspensions was determined by GFAAS (Perkin Elmer AAnalyst 600, Perkin Elmer, Shelton, USA). The silver standard solution (1000 mg/L in 5% HNO_3_) from Merck (Darmstadt, Germany) was used for calibration.

All prepared AgNPs were characterized by means of shape, primary size, size distribution, and surface charge in UPW. The particle visualization was performed by TEM (902A; Carl Zeiss Meditec AG, Jena, Germany) operated in bright-field mode with an acceleration voltage of 80 kV, while a Canon PowerShot S50 camera was used to capture the images. The sample preparation for TEM involved the deposition of a drop of the AgNP suspension onto a Formvar®-coated copper grid and air-drying it at room temperature. The TEM images were then used to measure the primary size (*d*, nm) of AgNPs for 100 particles per particle type by using the ImageJ software. The hydrodynamic diameter (*d*_H_) was determined by DLS at 25 °C using Zetasizer Nano ZS (Malvern Instruments, Malvern, UK) equipped with a green laser (532 nm) at an angle of 173°. The values of *d*_H_ were obtained as the value of the maximum peak of the size distribution by volume and reported as an average of ten measurements. The ζ potential values were determined by ELS using the same Zetasizer Nano ZS instrument. The values were calculated from the measured electrophoretic mobility and reported as an average of six measurements. The Zetasizer software (6.32; Malvern Instruments, Malvern, UK) was used for data processing.

### Experiments with animal tissues

Three-month-old male Wistar rats, 320–350 g (b.w.) were bred at the Unit for Laboratory Animals, at the Institute for Medical Research and Occupational Health, Zagreb, Croatia. The animals were kept under specific pathogen-free conditions, in polycarbonate cages with temperature (23 ± 2 °C) and humidity (55 ± 7%) control, with a 12 h light/dark reversed cycle. They were fed standard GLP-certified food (Mucedola, 4RF21, Italy) and given tap water ad libitum. Healthy rats were randomly selected and divided into control and treatment groups. The animals were treated by oral gavage with PVP-AgNPs at a daily dose of 1 mg Ag/kg b.w. during 28 days, while control animals were administered with the same amount (0.1 mL) of physiological solution. Despite the applied dose was much higher than the estimated amount of daily consumption of Ag by humans through ingestion (i.e. 20–80 µg) [61], the dose of 1 mg Ag/kg b.w. for animal experiments is 5–50 times lower than the doses tested in most in vivo studies [40].

After treatment the animals were sacrificed under general anaesthesia (Narketan, Vetoquinol UK Ltd., 80 mg/kg b.w.; Xylapan, Vetoquinol UK Ltd., 12 mg/kg b.w., intraperitoneal) following whole blood and tissue collection. The blood was collected by intracardiac puncture into a heparinised tube and the plasma was separated by centrifugation. The kidneys, liver, and brain were carefully removed and washed in ice-cold saline. Each tissue (1 g) was homogenised in 10 mL of PBS (0.05 M, pH 7.4) containing 0.1 mM of EDTA using a motor-driven homogenizer. Both WB and BP were diluted to 10% with PBS. AgNO_3_, PVP-AgNP, AOT-AgNP, and PLL-AgNP were mixed at a concentration of 100 mg Ag/L with 1% of WB, BP, and tissue homogenates for 30 min on a digital waving rotator (Thermo Scientific, USA). After incubation, the samples were diluted 1:5 with PBS, dropped on a Formvar®-coated TEM grid, air-dried, and examined by TEM (PBS was used as control).

In addition, liver tissues obtained from animals treated with PVP-AgNPs were prepared for TEM examination. The specimens were fixed in 2.5% glutaraldehyde in 0.1 M of PB, pH 7.4. After rinsing in the same buffer, the specimens were post-fixed in 2% osmium tetroxide in the same buffer for 30 min. All the specimens were then dehydrated in a series of graded alcohols, and embedded in TAAB embedding resin (TAAB, Aldermaston, UK). After sectioning on a Leica UC6 ultramicrotome (Leica Microsystems, Vienna, Austria) using a Diatome diamond knife (Ultra 45; Diatome, Biel, Switzerland), they were contrasted using uranyl acetate and lead citrate. The TEM images were made in a microscope (902A; Carl Zeiss Meditec AG, Jena, Germany) operating in bright-field mode with an acceleration voltage of 80 kV and a Canon PowerShot S50 camera was used to capture the images.

All experiments were performed following the animal welfare international standards and national legislation and were approved by the Animal Care Committee and Ethical Committee of the Institute for Medical Research and Occupational Health.

### Stability evaluation of AgNPs in artificial biological media

The behaviour and transformations of AgNPs were assessed after the exposure to different biological media (PB, PBS, CCM, m(CCM+BSA), mCYS, m(CYS+BSA), mGSH, m(GSH+BSA), ALF, and AGF), as well as in WB, BP, and 10% (v/v) homogenates of rat tissues (liver, brain, kidney) of male rats. The exact composition and parameters of each medium are listed in Table 1. The preparation of ALF followed a protocol published elsewhere [62] and included a pH adjustment to 4.5 with NaOH solution.

The stock suspensions of AgNPs (1000 mg Ag/L) in UPW were diluted in each medium (see Table 1) to a final AgNP concentration of 10 mg Ag/L and incubated at room temperature on a digital waving rotator (Thermo Scientific, USA), protected from light. The changes in size distribution and zeta potential were monitored by DLS and ELS, respectively, with the recordings taken at 0, 1, 4, and 24 h after mixing. Additionally, UV–vis spectra of all AgNPs suspensions in UPW and different biological media were recorded on a CARY 300 spectrophotometer (Varian Inc., Australia) following the same conditions. The UV–vis measurements were performed in a quartz cuvette with an optical path of 1 cm, in the wavelength range of 200–700 nm.

The dissolution behaviour of AgNPs was tracked in UPW, CCM, m(CCM+BSA), mCYS, m(CYS+BSA), mGSH, m(GSH+BSA), ALF, and AGF after 0, 1, 4, and of 24 h of incubation. Briefly, stock suspensions of AgNPs were diluted in the tested media to a final concentration of 10 mg Ag/L and kept in the dark at room temperature. A sample was taken immediately after dilution (0 h) and subsequently after 1, 4, and 24 h. The samples were processed by ultrafiltration using Amicon-4 Ultra centrifugal filter units with a cut-off size of 3 kDa (Merck Millipore, Darmstadt, Germany) in order to separate the dissolved silver from the AgNPs. The filtrates were immediately acidified with Suprapur HNO_3_ to final acid content of 10% (v/v). The quantification of dissolved Ag in the filtrates was performed by GFAAS (Perkin Elmer AAnalyst 600, Perkin Elmer, Shelton, USA) with Zeeman background correction. The results are presented as % of the dissolved Ag fraction compared to the total Ag content in AgNP suspensions before filtration.

Transmission electron microscopy was performed for different AgNPs dispersed in PBS, mCYS, and mGSH media at a concentration of 10 mg Ag/L after 30 min of incubation. The samples were diluted 1:5 with PBS, dropped on a Formvar®-coated copper grid, air-dried, and examined by TEM. The TEM visualization was also employed to inspect possible transformation of ionic Ag to AgNP in mCYS and mGSH media by dissolving AgNO_3_ in each media at a concentration of 100 mg Ag/L. After 2 h of incubation at room temperature, 10 μL of the reaction mixture aliquot was dropped on a TEM grid, air-dried, and examined by TEM. The images were taken using a 300 kV transmission electron microscope FEI Tecnai F30 (FEI Company, The Netherlands) equipped with a high angle annular dark field (HAADF) detector and enabled to work in scanning transmission (STEM) mode to perform EDX analysis.

### NMR experiments

As the incubation of ionic Ag in mGSH media showed the appearance of AgNPs (observed both visually and by TEM), the interaction between AgNO_3_ and GSH was evaluated by NMR spectroscopy. The spectra of free GSH (5 mM) and a mixture containing 5 mM of GSH and 10 mM of AgNO_3_ in PB after 2 h of incubation at 25 °C were obtained. The samples were prepared with the addition of D_2_O to a final concentration of 10% (v/v). The ^1^H NMR spectra were recorded on a Varian INOVA 400 spectrophotometer (Varian, PaloAlto, CA, USA) operating at 399.6 MHz. The chemical shifts were expressed in parts per million (ppm), referenced to the residual water signal. All spectra were recorded at 25 °C. The signal of the solvent was suppressed by using the PRESAT pulse sequence, available in the VnmrJ (4.2A) software.

## Supporting Information

This file shows three tables with values of hydrodynamic diameter, % volume distribution, zeta potential, and % of dissolved AgNPs immersed in different media.

File 1 Experimental parameters of AgNPs diluted in different biological media.
