# Supplementary material for: Fate and transformation of silver nanoparticles in different biological conditions
Source: Beilstein J Nanotechnol. 2021 Jul 7;12:665–79. doi: 10.3762/bjnano.12.53 (PMC8275868; doi:10.3762/bjnano.12.53)
Supplement: File 1 — Experimental parameters of AgNPs diluted in different biological media. [file Beilstein_J_Nanotechnol-12-665-s001.pdf]

BEILSTEIN JOURNAL OF NANOTECHNOLOGY

Supporting Information

for

Fate and transformation of silver nanoparticles in different biological conditions

Barbara Pem, Marija Ćurlin, Darija Domazet Jurašin, Valerije Vrček, Rinea Barbir, Vedran Micek, Raluca M. Fratila, Jesus M. de la Fuente and Ivana Vinković Vrček

Beilstein J. Nanotechnol. **2021,** *12,* 665–679. doi:10.3762/bjnano.12.53

Experimental parameters of AgNPs diluted in different biological media

License and Terms: This is a supporting information file under the terms of the Creative Commons Attribution License (https://creativecommons.org/licenses/by/4.0). Please note that the reuse, redistribution and reproduction in particular requires that the author(s) and source are credited and that individual graphics may be subject to special legal provisions.
The license is subject to the *Beilstein Journal of Nanotechnology* terms and conditions: (https://www.beilstein-journals.org/bjnano/terms)

Table S1: Hydrodynamic diameter and % volume distribution of AgNPs after 1, 4, and 24h in selected media, determined by DLS. The abbreviations of all media are explained in the Table 1 of the main manuscript.

Media	AOT AgNP			PVP AgNP			PLL AgNP		
	**1h**	**4h**	**24h**	**1h**	**4h**	**24h**	**1h**	**4h**	**24h**
DMEM	946.4 ± 20.3<br>(99%)	129.2 ± 55.8<br>(1%)<br>887.2 ± 152.7<br>(99%)	101.1 ± 10.1<br>(8%)<br>753.8 ± 148.4<br>(92%)	20.7 ± 0.0<br>(13%)<br>68.7 ± 11.7<br>(18%)<br>506.6 ± 141.2<br>(69%)	846.4 ± 99.8<br>(100%)	168.4 ± 75.0<br>(1%)<br>860.3 ± 262.6<br>(99%)	25.1 ± 0.0<br>(4%)<br>499.4 ± 135.0<br>(96%)	907.7 V 73.2<br>(100%)	98.0 ± 39.0<br>(4%)<br>635.1 ± 162.1<br>(93%)
DMEM + BSA	8.6 ± 2.3<br>(82%)<br>267.2 ± 43.4<br>(18%)	9.2 ± 3.7<br>(88%)<br>527.3 ± 144.5<br>(12%)	8.9 ± 3.4<br>(100%)	5.8 ± 1.6<br>(93%)<br>31.2 ± 4.2<br>(7%)	4.9 ± 1.4<br>(90%)<br>33.6 ± 2.3<br>(10%)	5.6 ± 1.3<br>(95%)<br>32.7 ± 5.4<br>(5%)	8.9 ± 2.8<br>(91%)<br>58.2 ± 4.8<br>(9%)	9.6 ± 4.2<br>(82%)<br>45.4 ± 5.7<br>(18%)	9.6 ± 2.2<br>(92%)<br>59.2 ± 7.8<br>(8%)
CYS	2.8 ± 0.5<br>(98%)<br>22.4 ± 2.6<br>(1%)	2.7 ± 0.7<br>(99%)<br>24.5 ± 1.4<br>(1%)	3.5 ± 0.3<br>(99%)<br>28.7 ± 4.2<br>(1%)	17.8 ± 0.0<br>(11%)<br>103.4 ± 4.6<br>(89%)	10.7 ± 3.7<br>(43%)<br>54.7 ± 0.0<br>(17%)<br>101.7 ± 9.4<br>(40%)	12.7 ± 0.1<br>(18%)<br>63.6 ± 5.7<br>(82%)	7.3 ± 1.4<br>(96%)<br>44.9 ± 6.2<br>(4%)	10.0 ± 2.9<br>(31%)<br>66.9 ± 28.1<br>(61%)<br>529.5 ± 208.7<br>(8%)	13.9 ± 0.9<br>(16%)<br>61.0 ± 10.8<br>(38%)<br>771.1 ± 205.1<br>(46%)
CYS + BSA	3.2 ± 0.1<br>(99%)<br>23.1 ± 1.9<br>(1%)	2.0 ± 0.5<br>(99%)<br>21.8 ± 1.4<br>(1%)	1.3 ± 0.0<br>(14%)<br>5.4 ± 2.5<br>(85%)<br>31.1 ± 12.5<br>(1%)	2.1 ± 0.6<br>(18%)<br>5.1 ± 0.9<br>(80%)<br>38.7 ± 3.2<br>(2%)	1.9 ± 0.0<br>(13%)<br>4.2 ± 0.6<br>(86%)<br>35.8 ± 0.8<br>(1%)	6.8 ± 0.6<br>(98%)<br>83.0 ± 7.2<br>(2%)	90.3 ± 3.8<br>(23%)<br>176.5 ± 24.8<br>(77%)	30.0 ± 0.5<br>(9%)<br>90.7 ± 3.4<br>(36%)<br>193.7 ± 21.7<br>(55%)	94.5 ± 0.0<br>(13%)<br>150.6 ± 21.0<br>(87%)
GSH	128.8 ± 2.6<br>(100%)	190.1 ± 3.3<br>(100%)	585.5 ± 74.8<br>(99%)	45.3 ± 0.0<br>(2%)<br>423.0 ± 45.0<br>(98%)	264.7 ± 5.6<br>(100%)	455.4 ± 17.9<br>(100%)	5.6 ± 1.6<br>(93%)<br>29.1 ± 11.8<br>(7%)	5.6 ± 1.6<br>(63%)<br>23.5 ± 6.6<br>(37%)	6.0 ± 0.8<br>(95%)<br>37.2 ± 3.5<br>(5%)
GSH + BSA	2.0 ± 0.0<br>(13%)<br>7.5 ± 1.0<br>(87%)	6.3 ± 0.9<br>(99%)<br>35.9 ± 0.0<br>(1%)	6.2 ± 0.6<br>(100%)	5.4 ± 0.1<br>(99%)<br>32.4 ± 0.0<br>(1%)	5.3 ± 0.4<br>(99%)<br>35.6 ± 0.0<br>(1%)	4.9 ± 0.2<br>(94%)<br>31.6 ± 0.5<br>(6%)	9.7 ± 2.8<br>(91%)<br>60.2 ± 51.8<br>(9%)	9.3 ± 2.2<br>(79%)<br>46.1 ± 6.7<br>(21%)	9.8 ± 3.2<br>(92%)<br>61.2 ± 8.4<br>(8%)
ALF	748.7 ± 97.5<br>(99%)	758.7 ± 103.5<br>(100%)	84.4 ± 19.0<br>(4%)<br>356.1 ± 141.2<br>(96%)	7.4 ± 1.2<br>(100%)	7.1 ± 0.9<br>(99%)<br>40.6 ± 6.4<br>(1%)	6.8 ± 2.6<br>(99%)<br>44.2 ± 0.0<br>(1%)	39.9 ± 12.6<br>(5%)<br>978.1 ± 28.0<br>(95%)	87.7 ± 9.2<br>(5%)<br>755.2 ± 120.5<br>(95%)	19.6 ± 0.0<br>(6%)<br>47.4 ± 7.8<br>(14%)

									363.7 ± 42.1 (80%)
AGF	811.5 ± 48.7 (99%)	762.7 ± 140.6 (100%)	644.0 ± 170.3 (100%)	9.2 ± 2.1 (99%)<br>48.0 ± 0.0 (1%)	3.9 ± 0.0 (13%)<br>10.6 ± 2.0 (80%)<br>70.6 ± 5.9 (7%)	16.0 ± 1.9 (31%)<br>87.2 ± 7.8 (69%)	4.4 ± 0.6 (86%)<br>30.7 ± 0.6 (14%)	7.1 ± 4.7 (42%)<br>636.2 ± 47.8 (58%)	4.8 ± 1.0 (18%)<br>29.9 ± 3.4 (42%)<br>617.6 ± 97.4 (40%)

Table S2: Zeta potential of AgNPs after 1, 4, and 24h in selected media determined by ELS. The abbreviations of all media are explained in the Table 1 of the main manuscript.

Media	AOT AgNP			PVP AgNP			PLL AgNP		
	1h	4h	24h	1h	4h	24h	1h	4h	24h
**DMEM**	−21.7 ± 0.6	−24.5 ± 0.7	−22.9 ± 1.1	−8.2 ± 0.4	−21.7 ± 0.5	−22.2 ± 0.8	24.8 ± 1.5	24.4 ± 1.8	23.3 ± 1.0
**DMEM + BSA**	−18.7 ± 2.4	−20.4 ± 3.7	−21.2 ± 3.2	−18.2 ± 4.1	−17.2 ± 3.8	−18.1 ± 2.6	−10.4 ± 4.2	−11.4 ± 1.7	−12.7 ± 2.5
**CYS**	−34.0 ± 2.4	−34.1 ± 1.3	−42.0 ± 1.9	−24.4 ± 0.6	−24.2 ± 1.2	−26.6 v 0.6	42.1 ± 2.9	32.1 ± 0.8	42.6 ± 3.2
**CYS + BSA**	−23.8 ± 2.4	−26.7 ± 3.0	−27.2 ± 2.1	−21.2 ± 0.6	−19.3 ± 1.4	−20.1 ± 2.3	−14.3 ± 1.1	−15.0 ± 0.5	−13.7 ± 2.8
**GSH**	−18.3 ± 1.7	−20.5 ± 1.5	−19.5 ± 0.5	−13.6 ± 0.1	−16.8 ± 0.5	−18.3 ± 0.3	26.9 ± 0.4	28.8 ± 0.7	28.0 ± 2.0
**GSH + BSA**	−10.2 ± 2.6	−16.1 ± 1.1	−15.5 ± 3.8	−8.9 ± 0.8	−10.3 ± 0.6	−9.9 ± 0.8	−10.3 ± 0.9	−10.3 ± 1.8	−12.2 ± 1.0
**ALF**	−16.9 ± 0.9	−19.4 ± 1.0	−16.6 ± 0.5	−8.6 ± 1.0	−10.9 ± 1.2	−9.2 ± 0.9	−8.3 ± 0.7	−8.1 ± 0.3	−8.3 ± 0.4
**AGF**	12.9 ± 1.9	12.4 ± 2.5	6.7 ± 0.4	0.1 ± 0.9	0.4 ± 1.5	0.6 ± 0.8	12.9 ± 1.3	10.3 ± 1.8	15.9 ± 1.0

Table S3. Percentages of Ag^{+} dissolved after 0, 1, 4, and 24 h of exposure of AgNPs to selected media. The abbreviations of all media are explained in the Table 1 of the main manuscript.

Media	AOT AgNP				PVP AgNP				PLL AgNP			
	0h	1h	4h	24h	0h	1h	4h	24h	0h	1h	4h	24h
**UPW**	0.8	1.0	1.9	3.0	0.6	0.7	1.0	1.3	0.4	0.8	0.9	1.3
**DMEM**	1.3	1.5	1.3	1.7	0.8	0.9	1.2	0.9	0.8	1.1	1.2	1.6
**DMEM + BSA**	0.5	0.7	0.8	0.6	0.4	0.6	0.7	1.0	0.6	0.7	0.7	0.9
**CYS**	0.5	0.4	0.4	0	0.3	0.2	0.2	0	0.2	0.1	0.1	0
**CYS + BSA**	0.3	0.4	0.5	0	0.2	0.3	0.5	0	0.4	0.2	0.2	0
**GSH**	0	0	0	0	0	0	0	0	0	0	0	0
**GSH + BSA**	0	0	0.1	0	0	0	0	0	0	0	0	0
**ALF**	2.1	1.2	2.0	1.5	0.8	0.7	0.9	0.9	1.6	2.3	2.0	1.7
**AGF**	2.5	2.3	2.2	1.5	1.1	1.3	1.5	1.4	2.1	2.2	2.4	2.2
